# Kernel principal component analysis and differential non-linear feature extraction of pesticide residues on fruit surface based on surface-enhanced Raman spectroscopy

**DOI:** 10.3389/fpls.2022.956778

**Published:** 2022-07-19

**Authors:** Guolong Shi, Xinyi Shen, Huan Ren, Yuan Rao, Shizhuang Weng, Xianghu Tang

**Affiliations:** ^1^School of Information and Computer, Anhui Agricultural University, Hefei, China; ^2^School of Electrical Engineering and Automation, Wuhan University, Wuhan, China; ^3^Key Laboratory of Agricultural Sensors, Ministry of Agriculture and Rural Affairs, Hefei, China; ^4^National Engineering Research Center for Agro-Ecological Big Data Analysis and Application, Anhui University, Hefei, China; ^5^Institute of Solid State Physics, Hefei Institutes of Physical Science (HFIPS), Chinese Academy of Sciences, Hefei, China

**Keywords:** surface-enhanced Raman spectroscopy, kernel principal component analysis, fruit pesticide residues, radial basis function, non-linear signal processing

## Abstract

Surface-enhanced Raman spectroscopy (SERS) has attracted much attention because of its high sensitivity, high speed, and simple sample processing, and has great potential for application in the field of pesticide residue detection. However, SERS is susceptible to the influence of a complex detection environment in the detection of pesticide residues on the surface of fruits, facing problems such as interference from the spectral peaks of detected impurities, unclear dimension of effective correlation data, and poor linearity of sensing signals. In this work, the enhanced raw data of the pesticide thiram residues on the fruit surface using gold nanoparticle (Au-NPs) solution are formed into the raw data set of Raman signal in the IoT environment of Raman spectroscopy principal component detection. Considering the non-linear characteristics of sensing data, this work adopts kernel principal component analysis (KPCA) including radial basis function (RBF) to extract the main features for the spectra in the ranges of 653∼683 cm^−1^, 705∼728 cm^−1^, and 847∼872 cm^−1^, and discusses the effects of different kernel function widths (σ) to construct a qualitative analysis of pesticide residues based on SERS spectral data model, so that the SERS spectral data produce more useful dimensionality reduction with minimal loss, higher mean squared error for cross-validation in non-linear scenarios, and effectively weaken the interference features of detecting impurity spectral peaks, unclear dimensionality of effective correlation data, and poor linearity of sensing signals, reflecting better extraction effects than conventional principal component analysis (PCA) models.

## Introduction

As a fungicide, thiram can effectively control apple scab and tomato rot, and is widely used in the cultivation of fruits and vegetables (Wang et al., 2019; Hussain et al., 2020; Gedam et al., 2021; Mbaye et al., 2022). Although the toxicity of formazan is relatively low, studies have shown that there are multiple potential harms from exposure to formazan. Currently, methods for the detection of agrochemical pollutants in fruits and vegetables include gas chromatography (Girard et al., 2021), high-performance liquid chromatography (Wei et al., 2021), gas chromatography-mass spectrometry (Ghatak et al., 2018), and liquid chromatography-mass spectrometry (Ye et al., 2020). Although these analytical techniques have good sensitivity for the quantitative detection of chemical pollutants, they still have shortcomings such as the inability to real-time monitoring, complicated operations, and cumbersome sampling process (Bereli et al., 2021). Therefore, it is necessary to propose simple and reliable methods to rapidly assess and detect pesticide residues on fruit surfaces. SERS is often used as a promising spectroscopic tool due to its advantages of high sensitivity, good specificity, simple preprocessing, and rapid spectral measurement (de Goes et al., 2019). At present, SERS has a wide range of analysis and applications and is often used to identify and detect chemical and biological species, as well as molecular imaging and monitoring at the cellular, tissue, and animal levels. It also has broad application prospects in the field of food safety (Abasi et al., 2020). Generally, SERS technology is a combination of Raman spectroscopy and nanoscience (Yoo et al., 2021), in which the molecules to be detected are adsorbed on or near the rough surface of transition metals, thereby increasing the Raman signal intensity in the local optical nanostructure region by several orders of magnitude. The effect is caused by the surface plasmon resonance of nanoparticles (Huang et al., 2020; Lin et al., 2020). As one of the most commonly used metal systems, gold nanoparticles (Au-NPs) are mainly used for SERS sensing (Zhang et al., 2017; Dowgiallo and Guenther, 2019; Szekeres and Kneipp, 2019).

Surface-enhanced Raman spectroscopy technology has great potential in detecting pesticide residues, but it still faces the following difficulties. First, good detection conditions are the basis for sensitive detection of SERS. At present, researchers have prepared various SERS substrates, but in the SERS detection of pesticide residues, they still lack high sensitivity, good repeatability, simple preparation, and low cost, which can not only enrich pesticide molecules, but also effectively enhance the suitability of the substrate (Kuo and Chang, 2014; Shen et al., 2022). In addition, there is still a lack of systematic research on the influence mechanism of different detection environments on pesticide SERS detection. Second, according to the electromagnetic enhancement mechanism, only molecules adsorbed or close to the surface of the substrate can undergo a plasmon resonance effect under light excitation, producing the SERS enhancement effect. And some pesticide molecules of weak affinity class can only produce a weak Raman signal or even no Raman signal (Krajczewski et al., 2020). In general, the SERS detection of pesticide residues on fruit surfaces is in the development stage. The ultra-sensitive detection based on SERS is prone to interference, so it is necessary to extract the characteristic information. The model of signal processing and signal recognition is still being explored, and a unified standard has not been formed. There are still many problems worth exploring.

In this work, SERS was used for the signal detection of thiram pesticide on the fruit surface, and the detection limits were determined by a combination of KPCA and partial least squares (PLS) chemometric methods after pre-processing with averaging, smoothing and differentiation, and finally, a model for the detection of thiram pesticide residues on the fruit surface was established to achieve qualitative and quantitative detection of thiram pesticide residues on the fruit surface, providing an experimental basis for the application of SERS to the detection of pesticide residues in fruits.

## Related works

Surface-enhanced Raman spectroscopy refers to the phenomenon that the molecules to be tested will be adsorbed on the surface of some solid metals (gold, silver, copper, etc.) or soil particles under the irradiation of incident light, resulting in the enhancement of their local electric field (Kim et al., 2019). The intensity of the Raman spectrum obtained at this time is 10^4^-10^6^ times higher than that of the ordinary Raman spectrum, which overcomes the defects of weak intensity and low sensitivity of the ordinary Raman spectrum (Zhan et al., 2019). SERS technology has also made great progress in detecting pesticide residues on the surface of fruits. Nanomaterials widely used in SERS detection of pesticide residues include noble metal sol substrates, mainly including gold, silver, and other nanoparticles, which can significantly enhance the SERS signal intensity of the analyte adsorbed on its surface (Xu et al., 2017; Ong et al., 2020). At present, the commonly used metal sol preparation methods include the electrochemical redox method, chemical deposition method, seed method, and so on, or else adding inducers such as NaC, NaNO_3_, and cysteamine hydrochloride to the metal sol can enrich the nanoparticles and generate a large number of hot spots to improve the enhancement effect (Krajczewski et al., 2020). Stamplecoskie et al. (2011) prepared silver nanoparticles (Ag NPs) by seed method and controlled their sizes, and detected 10^–3^ mol⋅L^−1^ rhodamine 6G (R6G), the results showed that the optimal size of Ag NPs was 50∼60 nm, the SERS intensity on the surface of R6G is the highest, and this method is expected to be extended to other adsorbents. Xu et al. (2017) developed a surfactant-free method to prepare popcorn-like Au-NPs for the detection of Chrysanthemum cicada on the peel surface. At present, SERS has been widely used in chemical science, biological science, safety, quality inspection, etc.

Generally, the dimension of independent variables is reduced in advance, and it is hoped that fewer features are used to express the original data, to make the constructed model simpler and the results more accurate and precise, and PCA is a widely used method. The rapid screening and identification of contaminants in food contact materials is another important approach with the help of data mining technology, among which, PCA has been widely adopted as a favorable tool for data mining (Liang et al., 2021). PCA can perform dimensionality reduction on big data so that useful information in the data can be quickly extracted and classified. At present, SERS combined with PCA has been used for the rapid detection of multiple targets such as multiple disease markers (Nargis et al., 2019), and good results have been achieved. Some scholars have used this method in combination with vector machines to propose a new method to solve the problem that the original model has a large amount of computation and a slow training speed when the data is high-dimensional. It is empirically found that the results of the new method are more accurate than methods such as neural networks.

Shin et al. (2018) demonstrated the correlation between non-small cell lung cancer (NSCLC) cell-derived exons and potential protein markers in cancer diagnosis through Raman scattering spectroscopy and PCA. Ai et al. (2018) analyzed the SERS spectrum of four different food colorants using modified PCA and identified characteristic bands. Uddin et al. (2021) proposed the use of variance accumulation for selecting top features from PCA data, from segmentally folded PCA (Seg Fol PCA) and spectrally segmented folded PCA (Seg Fol PCA) FE methods Intrinsic features are selected in the transformation space of, but the non-linear relationship between transformation features generated by the PCA-based finite element method cannot be exploited. KPCA operates on the covariance of non-linear transformations of the data, allowing a more flexible functional basis to be constructed. The basic idea of KPCA is to map the linearly no separable data in the low dimensional space to the higher dimensional space through some mapping function through the kernel function so that it can be linearly separable in the high dimensional space, and then use the relevant algorithms applicable to the linearly separable data for subsequent processing. When linear mapping may not get the desired results, KPCA has more advantages than PCA. Xin et al. (2020) used a kernel function to non-linearly map the calibrated samples to a high-dimensional space, evaluated the Raman spectral reconstruction accuracy based on the relative root mean square error, and reduced bad data and non-performing samples in the sample. Sun et al. (2019) proposed a model combining KPCA and support vector machine, which effectively eliminated the influence of noise in the spectrum. Wang et al. (2021) used the synthetic minority oversampling technique (SMOTE) to predict protein-protein interaction sites and applied KPCA to remove redundant features.

## Test principle and instrument reagents

### Mathematical expression of Raman signal

Many fields of physics, including plasma spectroscopy, atmospheric spectroscopy, nuclear physics, and nuclear magnetic resonance, can emit information-rich spectral lines whose contours approximate the Voigt function. The Voigt function is the result of the convolution of the Gaussian function and the Lorentzian function, and its calculation process is extremely complicated. Studies have shown that the Voigt peak function is divided into a Gaussian peak and a superposition of a Lorentz peak with the same center position and half-width, and its approximate form can be expressed as (Ejiri et al., 2021):


$$V\,\left(\nu \right)=\theta \,\alpha \,\exp\left[-\frac{4\,\ln2\,{\left(\nu -\omega \right)}^{2}}{\gamma^{2}}\right]+\left(1-\theta \right)\,\alpha \,\frac{\gamma^{2}}{{\left(\nu -\omega \right)}^{2}+\gamma^{2}}$$

In this equation, ν is the wave number, θ is the Gauss-Lorentz coefficient, α is the peak height, ω is the center position of the peak, and γ is the half-width of the peak. The spectral signal curve is formed by the superposition of dozens or hundreds of Voigt peaks. Tracing back to the source, the mathematical analysis of the vibrational spectral signal is to use the Voigt function to mathematically describe the spectral curve. The Voigt peak function is divided into a superposition of a Gaussian peak and a Lorentzian peak with the same center position and half-width. Therefore, the spectral peaks of the Raman and other vibrational spectra have the contour of the Lorentzian function, and its form can be expressed as:


$$L\,\left(v\right)=\frac{1}{\pi }\,\frac{\gamma_{L}^{2}}{{\left(v-w\right)}^{2}+\gamma_{L}^{2}}$$

In this equation, ν is the wave number, *γ_*L*_* is the half-width of the Lorentz peak, and ω is the center position of the peak. However, the spectrum is often affected by a variety of factors, such as altitude, air pressure, or the power distribution of the laser, and the Lorentz peak profile changes accordingly. Mathematically, the effects of these factors are generally approximated by the convolution of a Gaussian function. The following is the representation of the Gaussian function:


$$G\,\left(v\right)=\frac{1}{\gamma_{G}}\,{\left(\frac{\ln2}{\pi }\right)}^{1/2}\,\exp\left[-{\left(\frac{v-w}{\gamma_{G}}\right)}^{2}\,\ln2\right]$$

Where *γ_*G*_* is the half-width of the Gaussian peak.

The research shows that the Raman spectral signal obtained by the instrument is not only the real Raman spectrum but the result of the co-convolution of the real Raman spectrum showing the Lorentzian line shape and the instrument function showing the Gaussian line shape. The half-width of the latter depends on the resolution of the Raman spectrometer, and the half-width of the actual Raman spectral peak is much larger than the resolution of the Raman spectrometer. Therefore, if equation of G(v) is used to fit the Voigt line shape, a higher fitting accuracy can be obtained, which is suitable for various quantitative analysis situations.

### Raman spectrum testing instrument

**FIGURE 1:**
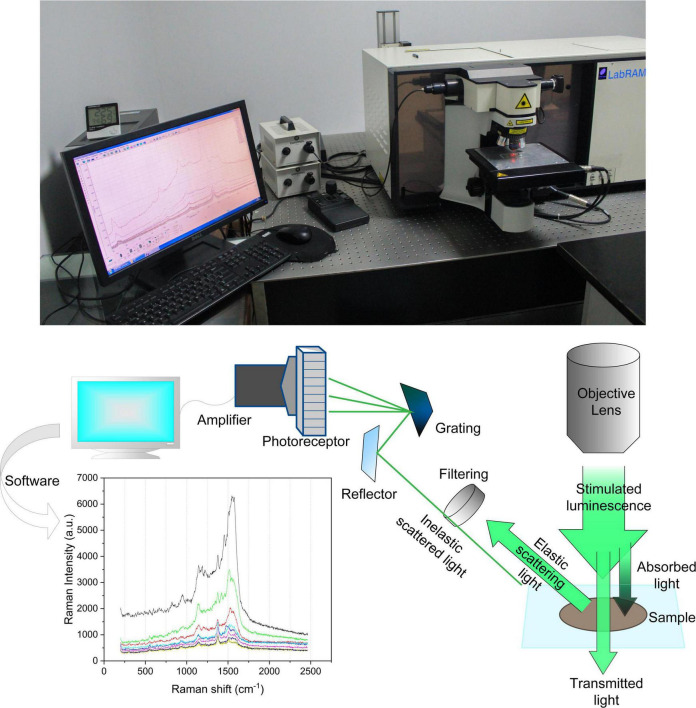
The IoT environment for the detection of principal components of pesticide residues on the surface of fruits by Raman spectroscopy.

The IoT environment for the detection of principal components of pesticide residues on fruit surfaces is shown in Figure 1. The Raman spectrometer used in the experiment is LabRAM-HR800 from HORIBA Jobin Yvon, France, and the specification model is Horiba Jobin-Yvon LabRAM-HR800. HR800 laser confocal Raman spectrometer has the function of *in situ* spectral research, overcomes the limitation that the original Raman spectrometer can only perform offline structural analysis of materials in an indoor open environment, and realizes non-destructive and non-invasive *in situ* measurement, which provides a reliable experimental technique for real-time monitoring of physical and chemical changes of substances under specific temperature, pressure, and atmosphere (Tang et al., 2015). The Raman spectrometer has a variety of laser wavelengths to choose from and can switch gratings automatically. The detection range is between 550 and 1550 cm^−1^ and the 633 nm laser used in this experiment is used as the Raman light source, dispersion system, and data processing system, which can meet the needs of data acquisition in this experiment.

The laser reaches the surface of the sample through a series of condensing lenses, mirrors, etc. In the focused state, the radiation power of the sample per unit area reaches the maximum. The laser-focused sample produces high energy and heat. Some biological samples or substances with lower melting points often need to reduce the power during testing. The six filters on the power attenuation wheel can achieve 1/2, 1/4, 1/10, 1/100, 1/1000, and 1/10000 six-gear power reduction. The dispersive system separates the Raman scattered light in space by wavelength, usually using a grating. An important parameter of the grating is the spectral resolution (R), which is a measure of the ability to separate two adjacent spectral lines at a specific wavelength (λ). That is, *R* = λ/△λ. The grating focal length (F) and the grating line density (N) are important factors to determine the spectral resolution (R), R∝F⋅N. The larger the F and N, the higher the spectral resolution. In addition, the spectral resolution is also related to the wavelength (λ). The larger the λ, the higher the spectral resolution. The powerful data analysis function is an indispensable part of an advanced Raman spectrometer. The Labspec 5 equipped with it has conventional data acquisition and analysis functions, and its imaging technology can generate images for different features of the spectrum (peak position, peak intensity), it also supports VB scripting language, and can also be used for Active X control in third-party applications, Labspec 5 software plug-ins can enter the spectral library and search, compatible with many commercial databases.

### Substrate preparation and data collection

**FIGURE 2:**
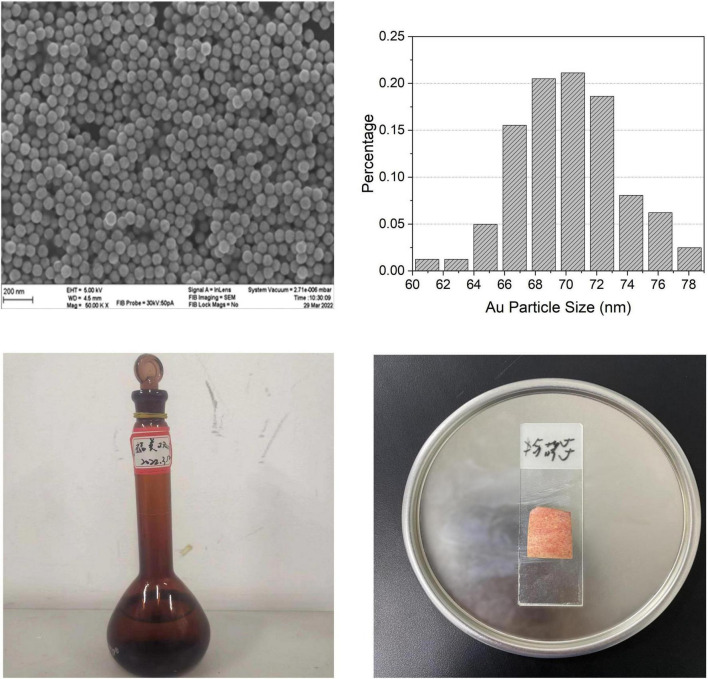
**(A)** Scanning electron microscope diagram of Au-NPs. **(B)** Particle diameter distribution diagram. **(C)** Preparation of thiram solution. **(D)** Dropping Au-NPs solution on the surface of the sample.

A 1.0 × 10^–4^ g⋅ml^−1^ solution sample of thiram solution was prepared in the laboratory, and the scanning electron microscope diagram of Au-NPs is shown in Figure 2A. It can be observed that the appearance of Au-NPs is spherical, and the particle diameter distribution diagram given in Figure 2B shows that its shape is relatively uniform. The specific operation was to dissolve 0.01 g of thiram sample in 100 ml of acetone, as shown in Figure 2C. Raman enhanced substrate is an important part of SERS technology. Au-NPs have stable properties and can generate local surface plasmon resonance under visible light irradiation. They are widely used in the preparation of reinforced substrates. The preparation method of Au-NPs is simple, the property is stable, and the reproducibility is high. It is an excellent material to strengthen the substrate. Therefore, 70 nm Au-NPs was prepared in the laboratory as the substrate for SERS detection (Wang et al., 2021). The test samples used in the experiment were the red Fuji apples purchased in the campus supermarket with basically the same size and weight, and Dangshan pears with almost the same size and weight, simulating the pesticide spraying process in the natural environment, that is, spraying on the surfaces of the two samples, respectively. The concentration of 1.0 × 10^–4^ g⋅ml^−1^ thiram, and then wait for 10-15 min after the fruit sample surface is automatically air-dried, take the sample epidermis, and then drop 5 microliters of Au-NPs solution on the sample epidermis, as shown in Figure 2D.

Limit of detection (LOD) refers to the corresponding amount of three times the instrument background signal generated by the matrix blank, or the average value of the background signal generated by the matrix blank plus three times the mean standard deviation. In the qualitative analysis of trace amounts, the LOD is used as the evaluation index to measure the enhancement effect of the substrate. For quantitative analysis, it is necessary to focus on uniformity and reproducibility. The substrate sensitivity and uniformity and repeatability have not yet reached a perfect balance. Sol-based substrates can achieve better detection limits and better SERS performance.

**FIGURE 3:**
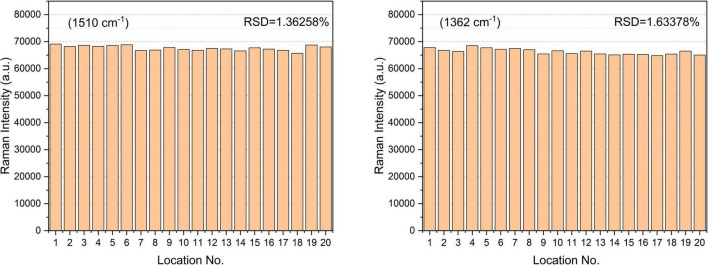
SERS spectral intensity of R6G (10^–4^ mol⋅L^–1^) measured at 20 positions on Au-NPs substrate.

Rhodamine 6G is a kind of dye that characterizes SERS. It has strong fluorescence and has a good application effect in SERS ultra-sensitive single-molecule detection. Rhodamine groups with blocked spironolactone units can produce cation-excited fluorescence and SERS signals. Their excellent photophysical properties are widely used in fluorescent probes and SERS. To explore the uniformity and repeatability of Raman enhancement of the Au-NPs substrate used in this work, the probe molecule R6G was selected for testing, and the probe molecules located at 1510 cm^−1^ (attributed to N-H in-plane bending) and 1362 cm^−1^ (attributed to C-H in-plane bending) were tested. Statistical calculation of the SERS peak intensity values (as shown in Figure 3) at the two peaks shows that the relative standard deviation (RSD) of the two peaks is only 1.36258 and 1.63378%, indicating that the prepared Au-NPs substrates have good homogeneity and reproducibility.

**FIGURE 4:**
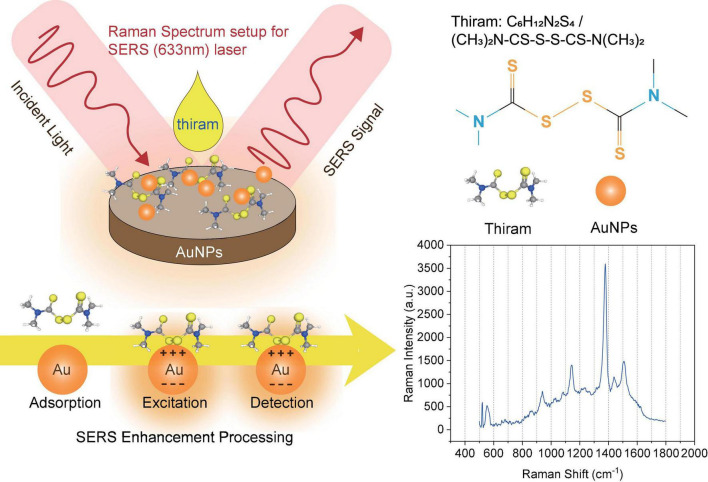
Schematic diagram of the plasma SERS model with enhancement effect with reaction time for the solution of thiram and Au-NPs.

During the experiment, thiram solution was sprayed on the sample for the first time and then detected by the Raman spectrum. The fluorescence signal and noise signal in the Raman spectrum experimental data obtained are very strong so that the characteristic peak signal of thiram solution is completely covered by interference, and the composition analysis of the data in the subsequent work cannot be completed. The edible wax on the skin of the fruit can not only keep it fresh but also prevent microorganisms from invading the fruit. There may be residual wax on the cleaned fruit surface, which causes a strong fluorescence signal to interfere with the Raman signal. Therefore, the experimental plan was improved in this work. The Au-NPs suspension was dropped on the tape with a pipette, and a drop was dropped every 8 cm or so, and it was left to stand for several hours until it was completely dry. Take different varieties of apples and pears and scrub the surface. And after drying, respectively, apply thiram solution on the surface of the fruit to air dry naturally. Adhere the tape coated with Au-NPs to the fruit containing thiram solution on the surface, peel it off after a few minutes, and place it on the Raman instrument detection table for detection (Liu et al., 2021). The transfer of pesticides to the tape can reduce the fluorescence signal and ensure the full reaction of pesticides with the substrate. Using a 633 nm light source and a 50× microscope, the LabSpec6 software collects data on different points on the surface of different varieties of apple and pear samples to obtain the raw data of the Raman spectrum on the surface of the sample. The obtained Raman spectrum data can be observed, as shown in Figure 4. The characteristic peak signal of the thiram solution indicates that the SERS can be used to obtain the original data of the sample surface. The original data includes the Raman spectra of Au-NPs on the tape, the Raman spectra of Au-NPs mixed with thiram on the fruit surface, the Raman spectra of thiram on the fruit surface, and the Raman spectra of thiram solutions with different concentrations.

## Signal processing and non-linear feature extraction

### Raman signal preprocessing

**FIGURE 5:**
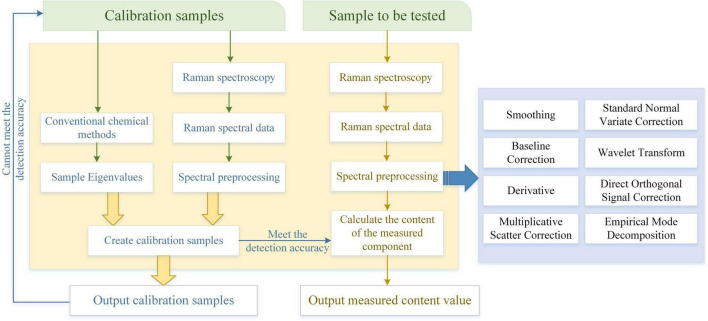
Flow chart of quantitative KPCA for SERS.

Since Raman scattering itself is relatively weak, the Raman spectrum is often affected by sample fluorescence, substrate fluorescence, natural light, and fluorescent light, resulting in high background and cosmic rays. When detecting the SERS signal of thiram, due to high-frequency random noise, fluorescence background, and sample unevenness, certain interference such as light scattering noise and baseline drift will be generated. The improvement of equipment often cannot eliminate these interference factors, and it is easy to affect the accuracy of subsequent prediction models. To obtain better experimental results, reduce noise, and improve the signal-to-noise ratio, the collected Raman signals must be analyzed. Perform certain preprocessing. Commonly used preprocessing methods include Smoothing, Baseline Correction, Derivative, Multiplicative Scatter Correction (MSC), and Standard Normal. Variate Correction (SNV), Wavelet Transform (WT), Direct Orthogonal Signal Correction (DOSC), and Empirical Mode Decomposition (EMD), are shown in Figure 5.

**FIGURE 6:**
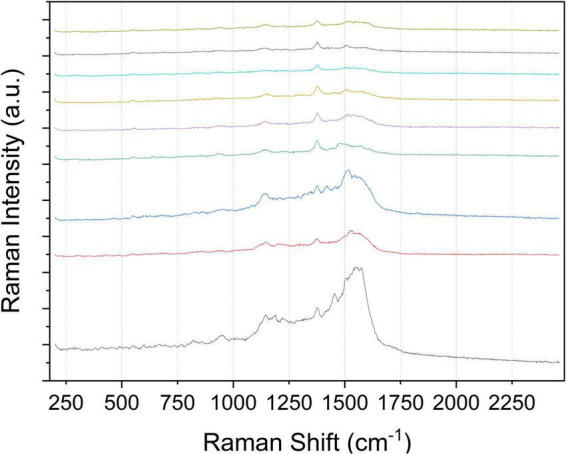
The spectrum after preprocessing the original Raman spectrum by subtracting the baseline, etc.

In this work, spectral averaging is used to average the SERS of thiram pesticides. Since the subsequent first-order differential and second-order differential processing will amplify the influence of noise, smoothing is used to remove the noise interference of the system and fluorescence. Improve the signal-to-noise ratio; finally, the overlapping peaks in the spectrum are separated by differential processing, and the first-order differential and second-order differential can, respectively, remove the drift that is independent of the same wavelength and linearly related. PLS is a regression modeling method of multiple dependent variables to multiple independent variables. By calculating the maximum variance between the spectral data and the target analyte, the relationship between the two is analyzed. It is suitable for complex multi-component Spectral analysis a widely used multivariate calibration method with good selectivity and predictive accuracy. PLS can eliminate the influence of data collinearity and effectively reduce the dimension of spectral data. After spectral averaging, smoothing, and differential processing, the implicit linear relationship between variables can be effectively detected due to the combination of appropriate chemometric methods, as shown in Figure 6. Therefore, the KPCA method and PLS method are used in this study to further construct the model to determine its non-linear relationship. To achieve the best fitting effect of the PLS model, the number of correction sets and prediction sets is very important. The experiment adopts the maximum-minimum strategy to establish a PLS model for samples according to a certain proportion of correction set and prediction set. First, calculate the average spectra of all candidate samples, and find the samples with the minimum and maximum distance from the average spectra to add to the calibration set. Then calculate the spectral distance between the remaining samples and each sample in the calibration set, find the samples with the minimum and maximum spectral distance from the average spectrum and add them to the calibration set, and repeat the above steps until the number of calibration sets reaches the set value, and the remaining samples are included in the prediction set.

### Non-linear kernel principal component analysis method

Surface-enhanced Raman spectroscopy spectrum is preprocessed to reduce noise interference and reduce or eliminate fluorescence background. However, because the SERS spectral data is up to thousands of dimensions and contains a lot of redundant information, the computational complexity of subsequent analysis increases, the accuracy rate decreases, and the model robustness is poor. To optimize the model and improve its prediction accuracy, the full spectrum variable modeling is usually not used, but the characteristic range spectrum is selected for analysis and processing, and the variables with a high contribution rate are extracted for modeling. Commonly used feature extraction methods include non-negative factorization (NMF), discrete cosine transform (DCT), PCA, etc. These methods obtain subject information in the sense of mathematical transformation after transforming the spectral signal.

Principal component analysis ignores the linear components with small variance and preserves the larger variance terms by processing the raw data. In this way, the dimension of effective data representation is reduced, the difficulty of problem processing is simplified, and the signal-to-noise ratio of data information is improved, to improve the prediction accuracy of the model. However, it usually requires the raw data to be Gaussian scores to extract better features, which greatly limits the practicality of this method. This is mainly because, in essence, the traditional PCA is a linear mapping method and does not do any non-linear processing, so it cannot detect the non-linear structure between the data. Therefore, many studies have extracted features between data by using non-linear PCA. On the other hand, an important feature of high-dimensional data is that the amount of data is huge, but the useful information that can be obtained from it is very limited, and there are different degrees of non-linear relationships. For this, traditional linear principal components are not sufficient.

Kernel principal component analysis uses an appropriate kernel function to project the original data space into a high-dimensional feature space. Generally, KPCA uses a non-linear kernel function to reconstruct a linear PCA, and the non-linear expansion of PCA can improve the dimensionality reduction quality of some non-linear data. KPCA maps the original data space to high-dimensional feature space and then performs PCA dimensionality reduction in the feature space.

Suppose the corresponding mapping is Φ, which is defined as Φ:*R^d^*→*F*,*x*→ξ = Φ(*x*). The kernel function is to implicitly realize the mapping from point *x* to *F* by mapping Φ, so that the data in the generated features satisfies the centralization condition, that is,


$$\sum_{\mu =1}^{M}\Phi \,\left(x_{\mu }\right)=0$$

The covariance matrix in the feature space is:


$$C=\frac{1}{M}\,\sum_{\mu =1}^{M}\Phi \,\left(x_{\mu }\right)\,\Phi \,{\left(x_{\mu }\right)}^{T}$$

The eigenvalues and eigenvectors can be obtained by solving, and the test sample projection in the eigenvector space *v^k^* is:


$$\left[\nu^{k}\cdot \Phi \,\left(x\right)\right]=\sum_{i=1}^{M}{\left(\alpha_{i}\right)}^{k}\,\left[\Phi \,\left(x_{i}\right),\Phi \,\left(x\right)\right]$$

Replacing the inner product with a kernel function, we have


$$\left[v^{k}\cdot \Phi \,\left(x\right)\right]=\sum_{i=1}^{M}{\left(\alpha_{i}\right)}^{k}\,K\,\left(x_{i},x\right)$$

When equation the above does not hold, it needs to be adjusted


$$\Phi \,\left(x_{\mu }\right)\to \Phi \,\left(x_{\mu }\right)-\frac{1}{M}\,\sum_{v=1}^{M}\Phi \,\left(x_{v}\right)\,\mu =1,\ldots ,M$$

Then the kernel matrix can be modified as


$$K_{\mu \,\nu }\to K_{\mu \,\nu }-\frac{1}{M}\,\left(\sum_{w=1}^{M}K_{\mu \,w}+\sum_{w=1}^{M}K_{w\,v}\right)+\frac{1}{M^{2}}\,\sum_{w,\tau =1}^{M}K_{w\,\tau }$$

The KPCA algorithm essentially extracts the non-linear structure of the original data through the non-linear transformation between the data space, feature space, and category space, and combines multiple related indices into several independent comprehensive indices, to reduce the dimension of the data and solve the problem of PCA in the processing of linearly inseparable data.

The kernel function K (kernel function) can directly obtain the inner product of the low-dimensional data mapped to the high-dimensional data, ignoring what the mapping function is, that is *K* < *x*,*y* > = < Φ(*x*), Φ(*y*)>, where x and y are low-dimensional input vectors, Φ is the mapping from low-dimensional to high-dimensional, and <*x*, *y*> is the inner product of *x* and *y*. Kernel functions provide a link from linear to non-linear and any algorithm that can represent only the dot product between two vectors. If we first map our input data to a higher-dimensional space, the effect of operations in this high-dimensional space will be non-linear in the original space. Commonly used kernel functions are Linear Kernel (Linear Kernel) *k*(*x*, *y*) = *x*^T^*y* + *c*, polynomial kernel(Polynomial Kernel) *k*(*x*, *y*) = (*ax*^T^*y* + *c*)*^d^*, Among them, the Radial Basis Function (Radial Basis Function) *k*(*x*, *y*) = exp(-γ||*x*-*y*||^2^), Also called Gaussian Kernel, because it can be one of the following kernel functions:


$$k\,\left(x,y\right)=\exp\left(-\frac{{||\text{x-y}||}^{2}}{2\,\sigma^{2}}\right)$$

The radial basis function refers to a real-valued function whose value only depends on the distance of a specific point, that is,


Φ⁢(x,y)=Φ⁢(||x-y||)

Any function Φ that satisfies the property is called a radial vector function, Standard generally uses Euclidean distance, although other distance functions are possible. Therefore, the other two commonly used kernel functions, the power exponential kernel and the Laplacian kernel, also belong to the radial basis kernel. In this work, the SERS spectrum including radial basis function (RBF) is used to extract the main features of the spectrum in the range of 653∼683, 705∼728, and 847∼872 cm^−1^, and the influence of different kernel function widths (σ) is discussed. Then, the support vector machine regression (SVR) algorithm was used to establish a regression model to predict the residues of thiram solution in the fruit epidermis, and the mean square error of interactive verification (RMSECV) was used to evaluate the performance of the model. The results are shown in Table 1.

**TABLE 1:** Predicted results of the model developed using chemometric methods.

Data	MLR	PLSR	KPCA + PLS	
	RMSECV/ (mg⋅L^−1^)	RMSECV/ (mg⋅L^−1^)	σ in KPCA	RMSECV/ (mg⋅L^−1^)
Spectra of	0.4507	0.4178	1000	3.902
653∼683,			5000	0.0347
705∼728,			8000	0.0305
847∼872 cm^−1^			10000	0.1828

It can be seen that the linear models built by multiple linear regression (MLR) and PLSR have higher RMSECV values, which may lead to lower accuracy of the prediction results; when σ is 1000, the prediction performance of the model built by KPCA combined with PLS is the worst, while the prediction performance improves when σ is 10000, but it is still weaker than when σ is 5000 and 8000. In conclusion, the model constructed by PCA combined with PLS with a σ of 8000 is the best. Its RMSECV is 0.0268 mg⋅L^−1^, the error is small, and it can accurately predict the residues of thiram solution.

The KPCA algorithm used is a qualitative and quantitative analysis model of pesticide residues written based on the measured SERS spectral data using Matlab software. The conversion equation of peak and pesticide concentration, through which qualitative and quantitative analysis of pesticide residues of unknown concentration can be carried out, and goodness of fit can be introduced to ensure that the error and accuracy of the model are within the allowable range. The goodness of fit refers to the fitting degree of the regression line to the observed value. The statistic to measure the goodness of fit is the determinate coefficient (also known as the determinate coefficient) r^2^. The maximum value of r^2^ is 1. The closer the value of r^2^ is to 1, the better the fitting degree of the regression line to the observed value is. Conversely, the smaller the value of r^2^, the worse the fitting degree of the regression line to the observed value.

### Peak attribution and principal component comparison of thiram solution

According to the molecular structure and conventional Raman spectra of thiram, thiram has obvious Raman characteristic peaks at 562, 929, 1146, 1379, and 1514 cm^−1^. The characteristic peak of 562 cm^−1^ is caused by S-S stretching vibration; the characteristic peak of 929 cm^−1^ is caused by C = S and C-N stretching vibration; the characteristic peaks of 1146 cm^−1^ and 1514 cm^−1^ can be attributed to C–N stretching and CH_3_ rocking vibration; the strongest characteristic peak at 1379 cm^−1^ is caused by the C-N stretching vibration and the CH_3_ symmetrical deformation vibration mode. Linear fitting was performed between the intensity (I) of the Raman peak at 1379 cm^−1^ and the concentration of the standard solution (N, μg⋅mL^−1^) of thiram, and the results showed that the mass concentration of thiram and the intensity of the Raman peak at 1379 cm^−1^ were linearly fitted. It has a good linear relationship. When the mass concentration range is 0.1∼5.0 μg⋅mL^−1^, it satisfies the linear regression equation I = 11644N + 4536.5 and the correlation coefficient *r*^2^ = 0.9912.

Compared with the standard Raman spectrum of thiram solution, the characteristic peaks of the Raman spectrum of the experimental sample data obtained by MATLAB are consistent with the standard Raman spectrum of thiram in the number of characteristic peaks and Raman displacement.

**FIGURE 7:**
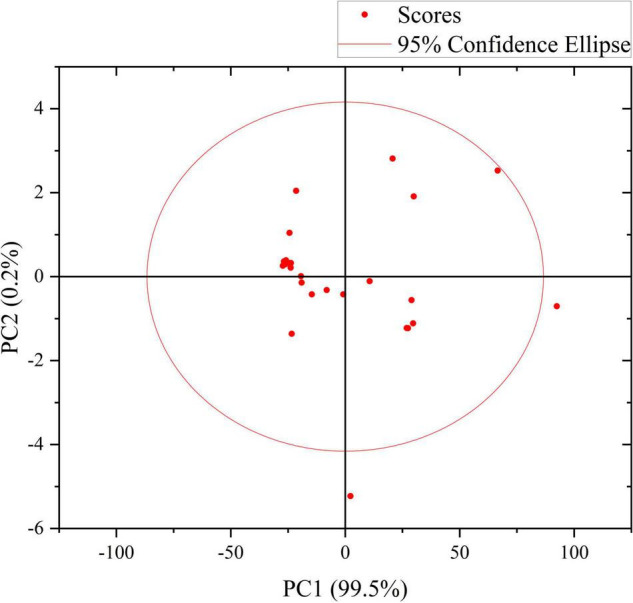
Score diagram of KPCA of thiram enhanced by Au-NPs solution.

In this work, by artificially applying the standard solution of thiram pesticide to the fruit samples that were not contaminated by the pesticide residues of thiram, the residual concentrations of thiram in the fruit epidermis were 0, 0.1, 0.5, 1.5, and 10 μg/g, respectively. Its SERS was measured under a Raman microscope, and each concentration was repeated four times, and the obtained SERS was smoothed, and baseline corrected. The characteristic peaks of the SERS in the water (1:1) solution are relatively consistent, and there are characteristic peaks at 750, 830, 1165, 1560 cm^−1^, etc., and the relationship between the intensity of the characteristic peak at 750 cm^−1^ and the concentration is the most obvious. Therefore, the characteristic peak at 750 cm^−1^ was selected to study the relationship between the peak intensity of the SERS and the concentration of fumes in the fruit epidermis. KPCA was used to determine the minimum detection limit of thiram in fruit epidermis. It can be seen from Figure 7 that the minimum detection limit of thiram in fruit epidermis is 0.1 μg/g, indicating that SERS can be used to detect thiram pesticide residues in fruit epidermis, and the minimum detection limit can reach 0.1 μg/g.

### Comparison with principal component analysis method

The visualization diagram of the 18 groups of sample data selected in this study. Through this diagram, we can compare it with the standard Raman spectrum of thiram solution after processing. From this, we can preliminarily determine whether the measured samples containing Pesticide residues are the characteristic peaks of the molecules of thiram, and with the graph of the experimental data, we can also see the similarity and difference of Raman spectra, even if the characteristic peaks of the same substance may not be the same, but the characteristics of the same substance The number of peaks is the same, and there is little difference between similar characteristic peaks. It can also be seen that the rapid detection of pesticide residues by KPCA Raman spectroscopy is more accurate.

**FIGURE 8:**
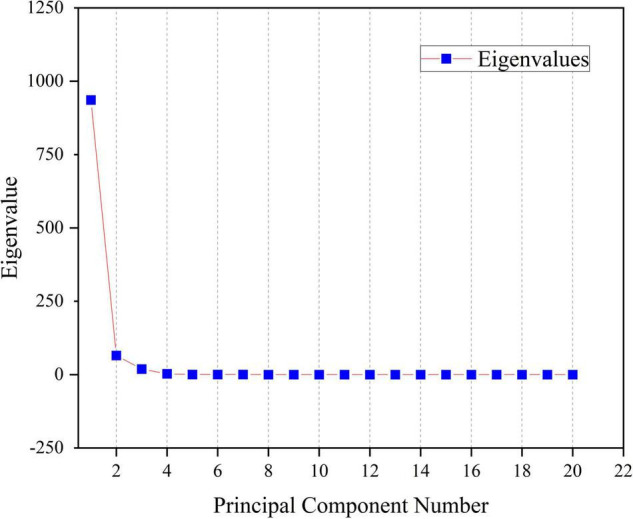
Relationship between the contribution of sample information and individual components.

**FIGURE 9:**
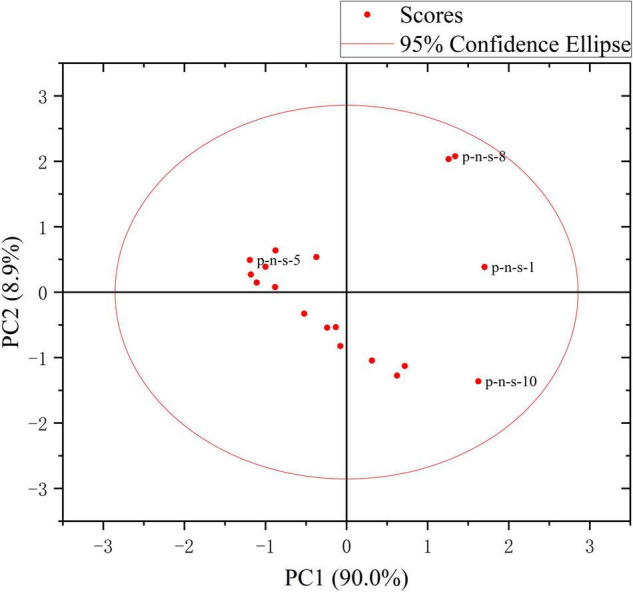
Two-dimensional scatter diagram of PC1 and PC2.

The first two principal components PC1 and PC2 have accounted for 98.9% of the variance. It can be seen from Figure 8 that the first principal component PC1 has explained most of the variance in the sample data matrix. Figure 9 shows a 2D scatter plot generated by PC1 and PC2, where the PC2 axis is perpendicular to the PC1 axis, which is often used for data classification. In Figure 9, it can be seen that the explained variance of PC1 for the experimental sample data is 90.0%, and the explained variance of PC2 is 8.9%. And the number of principal components shown in Figure 8 is also in full agreement with this data. It can be seen that Raman spectroscopy using KPCA combined with PLS is superior to the PCA model in terms of accuracy, precision, and stability.

## Conclusion

Ultra-sensitive detection based on SERS is prone to the interference of impurities and fluorescent substances. Therefore, to play the maximum role of SERS, it is necessary to extract feature information and establish a feature recognition model, that is, the recognition module of signal processing and signal recognition model for the relevant data spectrum library. In this work, by simulating the situation of pesticide residues on the fruit surface in the natural environment and based on the SERS detection technology, the pesticide residues on the fruit epidermis were determined. It was found that the metal particles in the SERS substrate could adsorb the pesticide components in the fruit epidermis. Thus, the Raman signal is enhanced, and the interference of the fluorescent signal and noise on the surface of the fruit is prevented to a certain extent. The performance of the models processed by non-linear kernel principal components is better than that of the models processed by principal components, which proves that the former has a better dimensionality reduction effect than the latter and makes the results more accurate. The probe molecule R6G was selected for comparative testing, and the relative standard deviation (RSD) of the two peaks was statistically calculated for the SERS peak intensity value, indicating that the prepared Au-NPs substrate had an excellent enhancement effect on pesticides. Then, with Au-NPs substrate as the enhancer, the Raman peaks of the standard product of thiram solution were compared, and the characteristic peaks for qualitative discrimination of thiram solution were determined based on the assignment of spectral peaks. Using the Raman spectroscopy technique based on KPCA, the punctuation samples are standardized and preprocessed, and then the samples are non-linearly mapped by the Gaussian kernel function. Non-linear factors improve the usability and operability of measurement data and reduce computational overhead. In this method, the substances in the Raman spectrum can be classified and the pesticide residues can be detected quickly. At the same time, this work is of great value to the practical popularization of SERS.

## Data Availability Statement

The raw data supporting the conclusions of this article will be made available by the authors, without undue reservation.

## Author contributions

GS was responsible for working as a supervisor for all procedures. XS was responsible for manuscript preparation and data processing. HR, SW, and YR participated in discussions and revisions. XT was responsible for providing the experimental platform and data collection. All authors contributed to the article and approved the submission.

## Conflict of Interest

The authors declare that the research was conducted in the absence of any commercial or financial relationships that could be construed as a potential conflict of interest.

## Publisher’s Note

All claims expressed in this article are solely those of the authors and do not necessarily represent those of their affiliated organizations, or those of the publisher, the editors and the reviewers. Any product that may be evaluated in this article, or claim that may be made by its manufacturer, is not guaranteed or endorsed by the publisher.
